# The advanced lung cancer inflammation index as a useful prognostic indicator for patients who underwent radical nephroureterectomy for upper tract urothelial carcinoma

**DOI:** 10.1007/s00345-025-05505-8

**Published:** 2025-02-20

**Authors:** Tomoya Hatayama, Keisuke Goto, Yuki Kohada, Kensuke Nishida, Takeshi Ueno, Tomoki Furutani, Kunihiro Hashimoto, Kenshiro Takemoto, Miki Naito, Shunsuke Miyamoto, Kohei Kobatake, Yohei Sekino, Hiroyuki Kitano, Akihiro Goriki, Keisuke Hieda, Nobuyuki Hinata

**Affiliations:** 1 Department of Urology, Hiroshima University Graduate School of Biomedical and Health Sciences, 1-2-3 Kasumi, Minamiku, Hiroshima, 734-8551 Japan; 2 Department of Urology, NHO Higashihiroshima Medical Center, Hiroshima, Japan; 3 Department of Urology, JA Hiroshima General Hospital, Hiroshima, Japan; 4 Department of Urology, NHO Kure Medical Center Chugoku Cancer Center, Hiroshima, Japan; 5 Department of Urology, JR Hiroshima Hospital, Hiroshima, Japan

**Keywords:** Inflammatory, Nutrition, Prognostic indicator, Radical nephroureterectomy, Upper urinary tract carcinoma

## Abstract

**Purpose:**

We aimed to evaluate the ability of the advanced lung cancer inflammation index (ALI) to predict the prognosis of patients who underwent radical nephroureterectomy (RNU) for upper tract urothelial carcinoma (UTUC). We also aimed to compare the ALI with other inflammatory or nutritional indices as prognostic indicators.

**Methods:**

We retrospectively evaluated patients who underwent RNU for UTUC at multiple centers between January 2010 and April 2024. We calculated the ALI before RNU and divided the patients into the low ALI and high ALI groups. We used 1:1 propensity score matching (PSM) to adjust the clinicopathological differences between two groups. We compared the overall survival (OS) and recurrence-free survival (RFS) of the low and high ALI groups using the Kaplan-Meier method. Furthermore, we assessed the ALI as a predictor of OS and RFS using a multivariate Cox proportional hazards regression analysis.

**Results:**

Of 488 patients (48.3% low ALI group), 160 patients from each group were matched. The Kaplan-Meier analysis revealed that the OS (*p* = 0.009) and RFS (*p* = 0.006) of the low ALI group were significantly shorter than those of the high ALI group. According to a multivariate analysis that included clinicopathological prognostic indicators, a low ALI was an independent predictor of poor OS (*p* = 0.014) and RFS (*p* = 0.038). Furthermore, according to the multivariate analysis including other inflammatory or nutritional indices, the ALI was an independent predictor of poor OS (*p* = 0.024) and RFS (*p* = 0.044).

**Conclusions:**

The ALI was a significantly useful prognostic predictors of patients with UTUC who underwent RNU.

**Supplementary Information:**

The online version contains supplementary material available at 10.1007/s00345-025-05505-8.

## Introduction

Upper tract urothelial carcinoma (UTUC) is rare and accounts for approximately 5% of all urothelial cancers [1, 2]. However, its incidence has increased in recent decades, likely because of advancements in detection methods and the aging population [3, 4]. Radical nephroureterectomy (RNU) is the standard treatment for localized UTUC, and especially for invasive and high-grade disease. UTUC is associated with the invasion of surrounding tissues and metastasis caused by the thinness of the ureteral wall and the prevalence of periductal lymphatic channels. Consequently, UTUC has a poor prognosis, with a 5-year survival rate of approximately 70%, even among patients who have undergone RNU [5].

Clinicopathological factors that can predict the prognosis of patients who underwent RNU for localized UTUC and stratify risk are age, ureteral tumors, hydronephrosis, high pathological stage, high disease grade, and lymphovascular invasion (LVI) [2]. Systemic inflammatory responses and the long-term nutritional status are well-established host-related mechanisms implicated in cancer development and progression [6]. Additionally, the neutrophil-to-lymphocyte ratio (NLR) [7], platelet-to-lymphocyte ratio (PLR) [8], systemic immune-inflammation index (SII) [9], and prognostic nutritional index (PNI) [10] have been suggested as prognostic factors for patients who underwent RNU for localized UTUC. The advanced lung cancer inflammation index (ALI), which is a novel biomarker of inflammation and nutrition, is a valid prognostic indicator of metastatic non-small cell lung cancer [11]. Moreover, the ALI is a prognostic indicator of not only metastatic disease but also localized cancer after radical surgery [12–14]. However, the efficacy of the ALI for localized UTUC has not yet been sufficiently assessed. The ALI is calculated based on the body mass index (BMI), serum albumin level, and NLR, and it can be used to comprehensively evaluate the inflammatory and nutritional statuses. In contrast, other indices, such as the NLR, PLR, SII, and PNI, reflect either the inflammatory status or the nutritional status. Therefore, we hypothesized that the ALI is associated with the prognosis of patients with localized UTUC and a strong predictor of poor prognoses. During this study, we evaluated the ability of the ALI to predict the prognosis of patients who underwent RNU for localized UTUC. We also compared the ALI with other inflammatory or nutritional indices.

## Methods

### Study design and patient selection

This retrospective study included Hiroshima University and its affiliated hospitals and was derived from the Hiroshima Cancer Registry Project (H-CARP). It was approved by the Ethics Committee of Hiroshima University in Japan (authorization number: E-2022-0003). Patients with UTUC who underwent laparoscopic RNU, including robotic surgery, between January 2010 and April 2024 were included. The exclusion criteria were as follow; patients who received neoadjuvant chemotherapy, with lymph or distant metastases before surgery, with hematological disorders and infections, and with incomplete clinicopathological characteristics. The patients data were managed using the Research Electronic Data Capture (REDCap) tools hosted at Hiroshima University [15, 16].

### Patients characteristics

The follow-up period, age, sex, BMI, neutrophil count, lymphocyte count, platelet count, serum albumin level, tumor location, hydronephrosis status, LND, pathological T stage, pathological N stage, histological grade, LVI, and adjuvant chemotherapy were extracted from the medical records and analyzed. Blood samples collected one month prior to undergoing RNU were used in the study. LND was performed at the discretion of the surgeon following the template of Kondo et al. [17]. Each inflammatory index or nutritional index was defined as follows: NLR = neutrophil count/lymphocyte count [7]; PLR = platelet count/lymphocyte count [8]; SII = platelet count (10^9^/L) × NLR [9]; PNI = 5 × lymphocyte count (10^9^/L) + (10 × serum albumin [g/dL]) [10]; and ALI = BMI × serum albumin (g/dL) / NLR [11]. The cut-off values for the NLR, PLR, SII, and PNI were determined based on a previous study [7–10].

### Outcomes

Recurrence-free survival (RFS) and overall survival (OS) were assessed to evaluate the prognosis. RFS was defined as the duration from RNU to local recurrence, lymph node metastasis, distant metastasis (not including intravesical or contralateral upper tract recurrence), or death. OS was defined as the time from RNU to death.

### Propensity score matching (PSM)

Propensity scores were calculated using multivariable logistic regression with the following variables: age, sex, tumor location, hydronephrosis status, histological grade, and LVI. Patients with low and high ALI were then matched 1:1 based on their propensity scores.

### Statistical analysis

All continuous variables are presented as the median (interquartile range [IQR]). Categorical variables are expressed as numbers and percentages. Each category was compared using Pearson’s chi-square test or the Wilcoxon signed-rank test. The RFS and OS rates were assessed using the Kaplan-Meier method. Factors that predicted RFS and OS were evaluated using univariate and multivariate Cox proportional hazards regression models. The cut-off values and area under the curve (AUC) of the ALI were determined using the receiver-operating characteristic curve analysis (Supplemental Fig. 1). Statistical significance was set at *p* < 0.05. All statistical analyses were conducted using JMP software (Pro 18 for Mac version 18.0.1; SAS Institute, Cary, NC, USA).

## Results

### Patient characteristics and the cutoff value of the ALI

We retrospectively reviewed 602 patients. After excluding 114 patients based on the exclusion criteria, a total of 488 patients were included in this study. The median follow-up period and age at the time of surgery were 30.9 months (IQR, 12.5–59.6 months) and 77 years (IQR, 70–82 years), respectively. Disease recurrence occurred in 128 patients and 88 patients died; for 53 patients who died, the cause of death was UTUC. The sensitivity, specificity, cut-off value, and AUC of ALI for OS at the final follow-up were 67.1, 55.8, 91.87, and 0.643 (Supplemental Table 1).

### Comparison of patient characteristics and pathological findings of the low and high ALI groups before and after PSM

We assigned 236 and 252 patients to the low and high ALI groups, respectively, based on an optimal cutoff value of 91.87. Before PSM, significant differences were observed between the groups in terms of follow-up period, age, sex, tumor location, hydronephrosis status, histological grade, and LVI. After PSM, there were 160 patients in each group, and no significant differences in clinicopathological prognostic indicators were observed between two groups (Table 1).

**Table 1:** Comparison of patients characteristics between ALI Low and high groups

		Before propensity score matching			After propensity score matching		
Variables	Overall Cases (*n* = 488)	ALI Low group (*n* = 236)	ALI High group (*n* = 252)	P-value	ALI Low group (*n* = 160)	ALI High group (*n* = 160)	P-value
Follow-up periods, months (IQR)	30.9 (12.5–59.6)	22.0 (9.0–48.4)	36.6 (17.1–61.3)	< 0.001	21.3 (8.0-51.4)	32.7 (14.4–55.4)	0.355
Age, < 75 / ≥75, n (%)	200 (41.0) /288 (59.0)	74 (31.4) /162 (68.6)	126 (50) /126 (50)	< 0.001	62 (38.7) /98 (61.3)	53 (33.1) /107 (66.9)	0.294
Gender, Male /Female, n (%)	344 (70.5) /144 (29.5)	147 (62.3) /89 (37.7)	197 (78.2) /55 (21.8)	< 0.001	110 (68.7) /50 (31.3)	107 (66.9) /53 (33.1)	0.719
BMI, (IQR)	22.9 (20.4–24.9)	21.8 (19.1–26.1)	23.7 (21.5–26.4)	< 0.001	21.8 (19.5–23.7)	23.6 (21.3–25.8)	< 0.001
Location, Ureter /Renal pelvis, n (%)	239 (49.0) /249 (51.0)	132 (55.9) /104 (44.1)	107 (42.5) /145 (57.5)	< 0.001	76 (47.5) /84 (52.5)	67 (41.9) /93 (58.1)	0.311
Hydronephrosis, n (%)	238 (48.8)	156 (66.1)	82 (32.5)	< 0.001	80 (50.0)	76 (48.2)	0.654
Lymph node dissection, n (%)	49 (10.0)	20 (8.5)	29 (11.5)	0.264	14 (8.8)	15 (9.4)	0.846
Pathological T stage, < 3 /≥3, n (%)	250 (51.2) /238 (48.8)	124 (52.5) /112 (47.5)	126 (50.0) /126 (50.0)	0.574	98 (61.3) /62 (38.8)	101 (63.1) /59 (36.9)	0.729
Pathological N positive, n (%)	3 (0.6)	1 (0.4)	2 (0.8)	0.597	1 (0.6)	2 (1.3)	0.558
Histological grade, 1–2 /3, n (%)	188 (38.5) /300 (61.5)	76 (32.2) /160 (67.8)	112 (44.4) /140 (55.6)	0.005	64 (40.0) /96 (60.0)	63 (39.4) /97 (60.6)	0.909
Lymphovascular invasion, n (%)	115 (23.7)	68 (29.1)	47 (18.7)	0.007	42 (26.3)	38 (23.8)	0.606
Adjuvant chemotherapy, n (%)	44 (9.7)	16 (7.4)	28 (11.7)	0.118	10 (6.8)	19 (12.6)	0.086
Neutrophil count, 10^9^/L (IQR)	3.76 (3.01–4.88)	4.28 (3.43–5.49)	3.30 (2.73–4.07)	< 0.001	4.18 (3.28–5.48)	3.29 (2.59–4.02)	< 0.001
Lymphocyte count, 10^9^/L (IQR)	1.56 (1.22–1.92)	1.27 (0.96–1.58)	1.79 (1.48–2.22)	< 0.001	1.19 (0.93–1.57)	1.81 (1.53–2.22)	< 0.001
Platelet count, 10^9^/L (IQR)	209 (175–255)	208 (172–254)	209 (178–255)	0.491	196 (168–256)	209 (175–254)	0.585
Serum albumin, g/dL (IQR)	4.1 (3.8–4.3)	4.0 (3.6–4.2)	4.2 (4.0-4.4)	< 0.001	4.0 3.6–4.2)	4.2 (4.0-4.4)	< 0.001
NLR, (IQR)	2.52 (1.83–3.37)	3.39 (2.82–4.32)	1.89 (1.50–2.34)	< 0.001	3.45 (2.86–4.49)	1.81 (1.48–2.26)	< 0.001
PLR, (IQR)	138.1 (107.9–175.2)	163.4 (132.3–218.3)	118.6(95.8–145.4)	< 0.001	169.6 (133.5-230.9)	117.6 (93.7-145.9)	< 0.001
SII, (IQR)	528.9 (371.1–747.4)	704.2 (530.2–1094.8)	396.1 (287.8–532.9)	< 0.001	707.7 (526.7-1140.9)	381.4 (271.4-521.2)	< 0.001
PNI, (IQR)	48.7 (45.5–52.4)	45.9 (42.3–51.6)	51.3 (48.5–54.0)	< 0.001	45.9 (42.4–48.8)	51.1 (48.2–53.5)	< 0.001
ALI, (IQR)	93.3 (66.3–93.3)	65.4 (46.2–79.4)	129.9 (105.7–177.1)	< 0.001	66.2 (46.3–79.6)	127.8 (104.6–173.0)	< 0.001

ALI, advanced lung cancer inflammation index; IQR, interquartile range; NLR, neutrophil-to-lymphocyte ratio; PLR, platelet-to-lymphocyte ratio; PNI, prognostic nutritional index; SII, systemic immune-inflammation index

### ALI is a novel predictor of OS and RFS after RNU

**Fig. 1:**
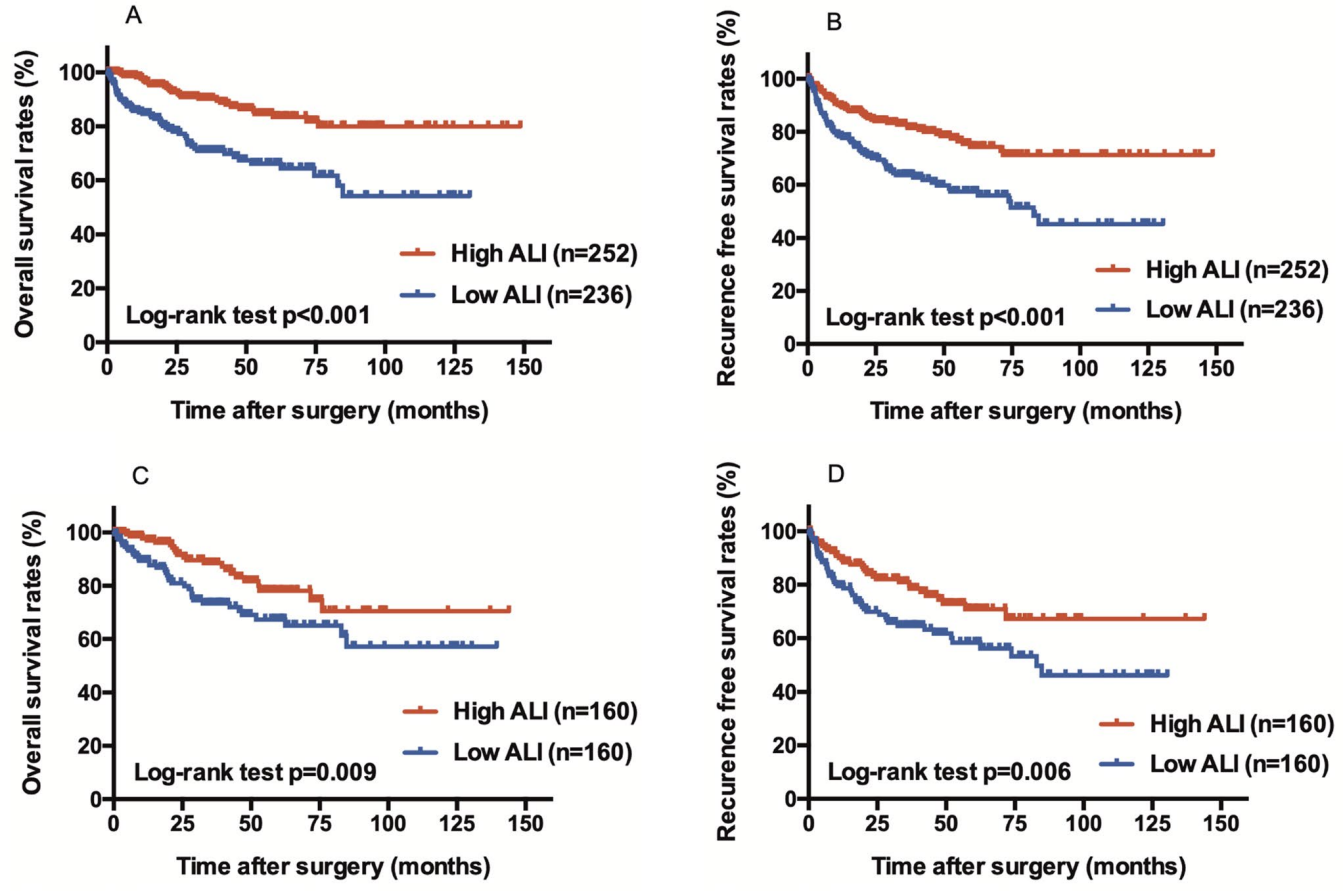
Survival curves comparing patients who underwent radical nephroureterectomy (RNU) between the high ALI group and the low ALI group, showing (**A**) overall survival (OS) before propensity score matching (PSM), (**B**) recurrence-free survival (RFS) before PSM, (**C**) OS after PSM, and (**D**) RFS after PSM. ALI, advanced lung cancer inflammation index

Before PSM, the Kaplan-Meier analysis revealed that the OS (*p* < 0.001) (Fig. 1A) and RFS (*p* < 0.001) (Fig. 1B) of the low ALI group were significantly shorter than those of the high ALI group. After PSM, the low ALI group continued to show significantly worse OS (*p* = 0.009) (Fig. 1C) and RFS (*p* = 0.006) (Fig. 1D) than the high ALI group. To determine the predictive factors for RFS and OS, we performed univariate and multivariate Cox proportional hazards regression analyses of the ALI and clinicopathological prognostic indicators (Table 2). The multivariate analysis demonstrated that age (75 years or older; hazard ratio [HR], 2.50; *p* < 0.001), high histological grade (HR, 2.07; *p* = 0.016), positive LVI (HR, 2.09; *p* = 0.006), and low ALI (HR, 1.95; *p* = 0.014) were significant independent predictors of shorter OS. Additionally, the multivariate analysis of RFS revealed that age (75 years or older; HR, 1.53; *p* = 0.036), pT stage (3≤; HR, 1.55; *p* = 0.045), high histological grade (HR, 1.80; *p* = 0.013), positive LVI (HR, 2.44; *p* < 0.001), and low ALI (HR, 1.58; *p* = 0.038) were significant independent predictors of poor RFS.

**Table 2:** Univariate and multivariate analyses including clinicopathological prognostic predictors and ALI of predictive factors for overall survival and recurrence free survival

	Overall Survival				Recurrence Free Survival			
	Univariate		Multivariate		Univariate		Multivariate	
	HR (95% CI)	P value	HR (95% CI)	P value	HR (95% CI)	P value	HR (95% CI)	P value
Age (≥ 75 vs. <75)	1.89 (1.30–2.76)	< 0.001	2.50 (1.48–4.24)	< 0.001	3.01 (1.85–4.95)	< 0.001	1.53 (1.03–2.27)	0.036
Gender (Male vs. Female)	0.96 (0.61–1.51)	0.854	1.07 (0.65–1.77)	0.773	0.88 (0.61–1.29)	0.532	0.95 (0.64–1.42)	0.814
Location (Ureter vs. Renal pelvis)	1.01 (0.71–1.43)	0.962	1.50 (0.92–2.45)	0.103	1.12 (0.74–1.71)	0.583	1.34 (0.89–2.00)	0.159
Hydronephrosis	2.15 (1.50–3.08)	< 0.001	1.44 (0.82–2.54)	0.199	2.60 (1.67–4.07)	< 0.001	1.33 (0.85–2.09)	0.211
Pathological T stage (≥ 3 vs. <3)	2.18 (1.51–3.15)	< 0.001	1.23 (0.73–2.08)	0.443	1.56 (1.02–2.40)	0.041	1.55 (1.01–2.37)	0.045
Histological grade (3 vs. 1–2)	2.84 (1.86–4.32)	< 0.001	2.07 (1.15–3.73)	0.016	3.08 (1.83–5.18)	< 0.001	1.80 (1.13–2.86)	0.013
Lymphovascular invasion	4.19 (2.96–5.95)	< 0.001	2.09 (1.23–3.54)	0.006	3.53 (2.31–5.37)	< 0.001	2.44 (1.59–3.74)	< 0.001
Adjuvant chemotherapy	2.59 (1.64–4.09)	< 0.001	1.27 (0.66–2.44)	0.474	2.01 (1.13–3.58)	0.017	1.29 (0.76–2.19)	0.339
ALI (Low vs. High)	2.77 (1.78–4.33)	< 0.001	1.95 (1.15–3.31)	0.014	2.17 (1.51–3.10)	< 0.001	1.58 (1.03–2.42)	0.038

ALI, advanced lung cancer inflammation index, CI, confidence interval; HR, hazard ratio

Furthermore, we compared the ALI with other inflammatory or nutritional indices as prognostic factors by performing univariate and multivariate Cox proportional hazards regression analyses (Table 3). According to the multivariate analysis, the ALI was an independent prognostic indicator of shorter OS (HR, 2.09; *p* = 0.024) and RFS (HR, 1.71; *p* = 0.044) with highest HR.

**Table 3:** Univariate and multivariate analyses including inflammatory or nutritional index of predictive factors for overall survival and recurrence free survival

	Overall Survival				Recurrence Free Survival			
	Univariate		Multivariate		Univariate		Multivariate	
	HR (95% CI)	P value	HR (95% CI)	P value	HR (95% CI)	P value	HR (95% CI)	P value
NLR (≥ 2.7 vs. <2.7)	2.05 (1.34–3.12)	< 0.001	1.14 (0.62–2.09)	0.669	1.83 (1.29–2.59)	< 0.001	1.22 (0.74–2.02)	0.443
PLR (≥ 126.88 vs. <126.88)	1.08 (0.71–1.65)	0.707	0.68 (0.41–1.10)	0.116	1.22 (0.85–1.73)	0.278	0.86 (0.57–1.30)	0.478
SII (≥ 550 vs. <550)	1.47 (0.96–2.23)	0.073	1.00 (0.58–1.74)	0.986	1.37 (0.97–1.94)	0.074	0.93 (0.59–1.47)	0.771
PNI (< 46.91 vs. ≥46.91)	2.65 (1.74–4.04)	< 0.001	2.02 (1.26–3.23)	0.003	1.93 (1.36–2.74)	< 0.001	1.49 (1.01–2.22)	0.046
ALI (Low vs. High)	2.77 (1.78–4.33)	< 0.001	2.09 (1.10–3.96)	0.024	2.17 (1.51–3.10)	< 0.001	1.71 (1.01–2.88)	0.044

ALI, advanced lung cancer inflammation index, CI, confidence interval; HR, hazard ratio; NLR, neutrophil-to-lymphocyte ratio; PLR, platelet-to-lymphocyte ratio; PNI, prognostic nutritional index; SII, systemic immune-inflammation index

## Discussion

For patients with localized UTUC who underwent RNU, a low ALI was significantly associated with older age, hydronephrosis, high histological grade, positive LVI, and poor OS and RFS. The ALI is a valid prognostic indicator of metastatic non-small cell lung cancer [11]. Then, the ALI can be used to assess the host-related inflammatory and nutritional statuses. Aging, renal dysfunction caused by hydronephrosis and other causes, and advanced cancer are associated with severe inflammation or malnutrition [6, 18–20]. Therefore, a low ALI is associated with other well-established predictors of poor prognoses, such as older age, presence of hydronephrosis, high histological grade, and positive LVI. Furthermore, after PSM, the low ALI group continued to show significantly worse OS and RFS compared to the high ALI group, despite the absence of significant differences in clinicopathological prognostic predictors between the two groups. Moreover, according to the multivariate Cox proportional hazards regression analysis that included clinicopathological prognostic indicators, a low ALI was a significant independent predictor of shorter OS and RFS. Therefore, our findings may help predict the prognosis of patients with localized UTUC before RNU. Additionally, this is the first study to evaluate the ALI as a biomarker for UTUC.

The inflammatory and nutritional statuses of the host are closely associated with the cancer prognosis [6]. Although several inflammatory or nutritional indices can be used to predict the prognosis of patients with UTUC and other cancers [7–10, 21–24], the ALI was most useful predictor of poor OS in our cohort. The ALI was calculated using the BMI, albumin level, and NLR. The BMI can be used to assess obesity and reflects the nutritional status of the host. Furthermore, studies have shown that a high BMI is associated with low systemic inflammation and a good prognosis; this phenomenon is known as the obesity paradox [25, 26]. Albumin reflects the nutritional status, and malnutrition decreases the serum albumin level. Cytokines such as IL-6 produced by tumors or surrounding cells decrease the production of albumin by hepatocytes, and inflammation decreases the serum albumin level [27]. The NLR is an easily accessible and cost-effective biomarker that can be determined using the standard complete blood count; additionally, it is a known systemic biomarker of inflammation related to cancer [21]. The ALI is determined using physical characteristics and blood test results; therefore, it may reflect the complex inflammatory and nutritional statuses of patients more accurately than other nutritional or inflammatory indices. Therefore, the ALI may be a stronger prognostic indicator than other nutritional or inflammatory indices. Furthermore, the factors that comprise the ALI are generally assessed before RNU; therefore, the ALI can be easily calculated and used in clinical practice.

UTUC has a poor prognosis, with a 5-year survival rate of approximately 70%, even for patients who have undergone RNU. The ability of multidisciplinary treatments, such as neoadjuvant chemotherapy, LND, and adjuvant therapy, to improve the oncological outcomes and, subsequently, the prognosis of patients with cancer has been validated; these treatments decrease disease recurrence and mortality rates [28–31]. Although multidisciplinary treatments comprising these modalities may improve the prognosis of patients with high-stage and high-grade UTUC, they could result in overtreatment for patients with low-stage and low-grade disease. Currently, there are no effective protein biomarkers for UTUC, and it is difficult to accurately diagnose the T stage before RNU. Therefore, selecting appropriate patients to receive multidisciplinary treatments before surgery is important; however, there are no useful criteria. The establishment of preoperative biomarkers, such as ALI, may lead to the development of criteria for these treatments. Thus, further studies are needed to evaluate the role of the ALI in clinical practice.

This study has several limitations. First, as a retrospective study conducted across multiple centers, the criteria for RNU, multidisciplinary therapy, pathological assessment, and follow-up schedules were not standardized. Additionally, the collection of comprehensive patient data, including comorbidities and smoking history, was limited due to incomplete records, patient deaths, and loss to follow-up. Selection bias and unmeasured confounding factors may also have been present. Second, the follow-up periods were too short to assess long-term prognosis. Third, standardized cut-off values for other inflammatory and nutritional indices have not yet been established. These limitations may have influenced the results of our study. While further research is needed to refine the optimal cut-off value and definitively establish the role of ALI in predicting survival, this is the first study to reveal the efficacy of the ALI for patients who underwent RNU for localized UTUC.

## Conclusions

Our study demonstrated that, compared to a high ALI, a low ALI was significantly associated with shorter OS and RFS of patients with localized UTUC who underwent RNU. According to the multivariate Cox proportional hazard regression analysis that included well-established clinicopathological prognostic indicators and other nutritional or inflammatory indices, the ALI is an independent prognostic indicator. Therefore, our findings revealed the efficacy of the ALI as a prognostic indicator that may help with decisions regarding perioperative treatment options.

## Electronic supplementary material

Below is the link to the electronic supplementary material.

Supplementary Material 1: The receiver-operating characteristic curve of the advanced lung cancer inflammation index (ALI). AUC, area under the curve; CI, confidence interval.

Supplementary Material 2

## Data Availability

No datasets were generated or analysed during the current study.
