# Supplementary figures and images for: The advanced lung cancer inflammation index as a useful prognostic indicator for patients who underwent radical nephroureterectomy for upper tract urothelial carcinoma
Source: World J Urol. 2025 Feb 20;43(1):132. doi: 10.1007/s00345-025-05505-8 (PMC11842494; doi:10.1007/s00345-025-05505-8)

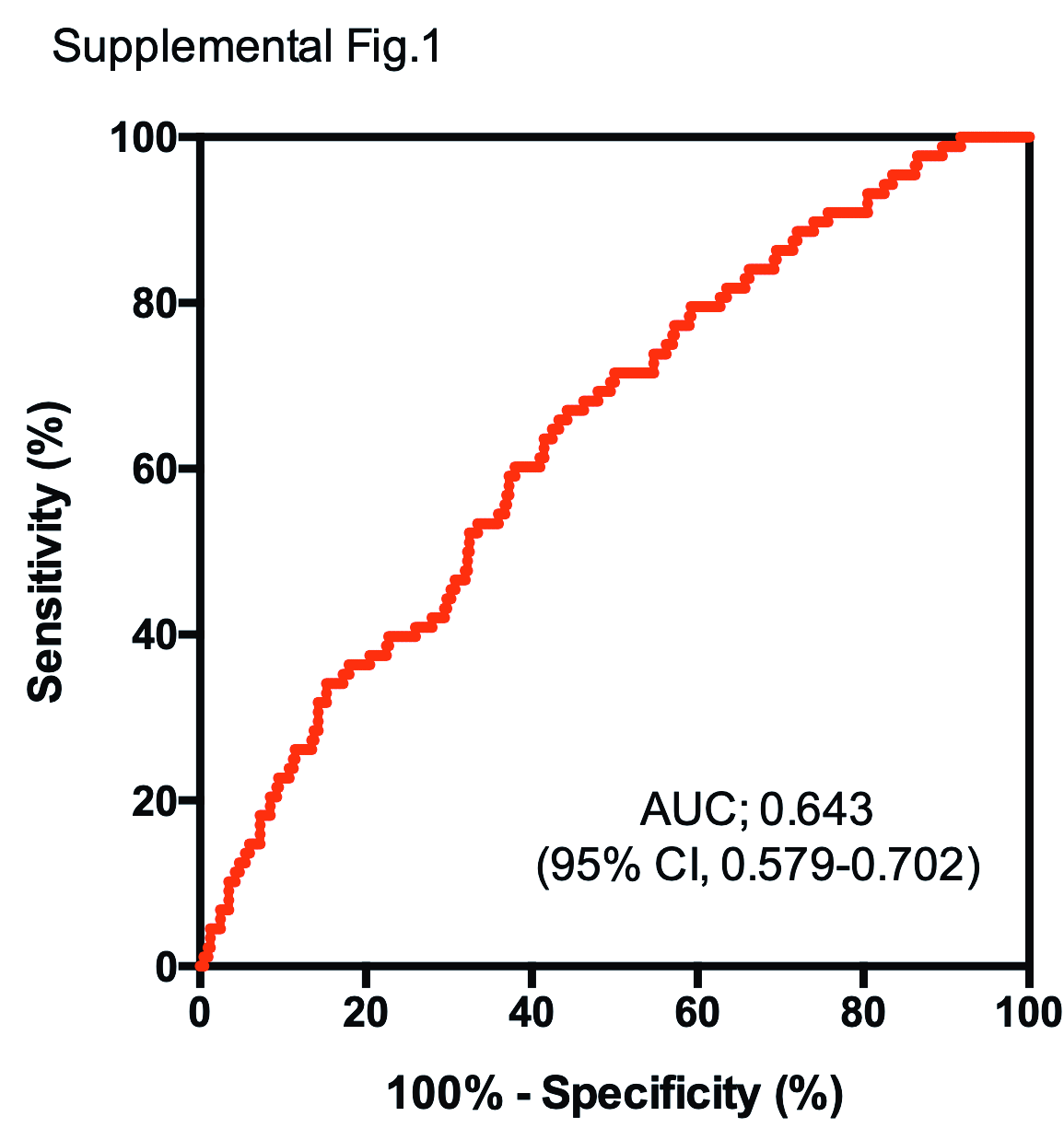

Supplement: Supplementary file 1 — Supplementary Material 1: The receiver-operating characteristic curve of the advanced lung cancer inflammation index (ALI). AUC, area under the curve; CI, confidence interval. [file 345_2025_5505_MOESM1_ESM.tiff]
