# Supplementary material for: The advanced lung cancer inflammation index as a useful prognostic indicator for patients who underwent radical nephroureterectomy for upper tract urothelial carcinoma
Source: World J Urol. 2025 Feb 20;43(1):132. doi: 10.1007/s00345-025-05505-8 (PMC11842494; doi:10.1007/s00345-025-05505-8)
Supplement: Supplementary file 2 — Supplementary Material 2 [file 345_2025_5505_MOESM2_ESM.docx]

| Supplemental Table 1: Sensitivity and specificity at the cut-off value predicting overall survival in inflammation or nutritional indices | | | | | |
| --- | --- | --- | --- | --- | --- |
| Variables | OS sensitivity (%) | OS specificity (%) | Cut-off value | AUC | 95% CI |
| NLR | 50.0 | 68.3 | 2.94 | 0.611 | 0.544-0.673 |
| PLR | 12.5 | 92.3 | 262.4 | 0.491 | 0.423-0.559 |
| SII | 47.7 | 64.0 | 62.1 | 0.548 | 0.479-0.614 |
| PNI | 44.3 | 79.0 | 45.48 | 0.633 | 0.564-0.697 |
| ALI | 67.1 | 55.8 | 91.87 | 0.643 | 0.579–0.702 |
| ALI, advanced lung cancer inflammation index; AUC, area under curve; CI, confidence interval; IQR, interquartile range; NLR, neutrophil-to-lymphocyte ratio; OS, overall survival; PLR, platelet-to-lymphocyte ratio; PNI, prognostic nutritional index; SII, systemic immune-inflammation index | | | | | |
